# Salivary gland *LAMP3* mRNA expression is a possible predictive marker in the response to hydroxychloroquine in Sjögren’s disease

**DOI:** 10.1371/journal.pone.0282227

**Published:** 2023-02-23

**Authors:** Hiroyuki Nakamura, Tsutomu Tanaka, Youngmi Ji, Changyu Zheng, Sandra A. Afione, Blake M. Warner, Fabiola Reis Oliveira, Ana Carolina F. Motta, Eduardo M. Rocha, Masayuki Noguchi, Tatsuya Atsumi, John A. Chiorini

**Affiliations:** 1 Adeno-Associated Virus Biology Section, National Institute of Dental and Craniofacial Research, National Institutes of Health, Bethesda, MD, United States of America; 2 Salivary Disorder Unit, National Institute of Dental and Craniofacial Research, National Institutes of Health, Bethesda, MD, United States of America; 3 Department of Clinical Medicine, Ribeirão Preto Medical School, University of São Paulo, São Paulo, Brazil; 4 Department of Stomatology, Public Health and Forensic Dentistry, School of Dentistry of Ribeirão Preto, University of São Paulo, São Paulo, Brazil; 5 Department of Ophthalmology, Otorhinolaryngology, Head and Neck Surgery, Ribeirão Preto Medical School, University of São Paulo, São Paulo, Brazil; 6 Division of Cancer Biology, Institute for Genetic Medicine, Hokkaido University, Sapporo, Japan; 7 Department of Rheumatology, Endocrinology and Nephrology, Faculty of Medicine, Hokkaido University, Sapporo, Japan; Faculty of Medicine, University of Belgrade, SERBIA

## Abstract

Hydroxychloroquine (HCQ) is a lysosomotropic agent that is commonly used for treating Sjögren’s disease (SjD). However, its efficacy is controversial because of the divergent response to the drug among patients. In a subgroup of SjD patients, lysosome-associated membrane protein 3 (LAMP3) is elevated in expression in the salivary glands and promotes lysosomal dysregulation and lysosome-dependent apoptotic cell death. In this study, chloroquine (CQ) and its derivative HCQ were tested for their ability to prevent LAMP3-induced apoptosis, *in vitro* and on a mouse model of SjD. In addition, efficacy of HCQ treatment was retrospectively compared between high *LAMP3* mRNA expression in minor salivary glands and those with *LAMP3* mRNA levels comparable with healthy controls. Study results show that CQ treatment stabilized the lysosomal membrane in LAMP3-overexpressing cells via deactivation of cathepsin B, resulting in decreased apoptotic cell death. In mice with established SjD-like phenotype, HCQ treatment also significantly decreased apoptotic cell death and ameliorated salivary gland hypofunction. Retrospective analysis of SjD patients found that HCQ tended to be more effective in improving disease activity index, symptom severity and hypergammaglobulinemia in patients with high *LAMP3* expression compared those with normal *LAMP3* expression. Taken together, these findings suggested that by determining salivary gland *LAMP3* mRNA level, a patient’s response to HCQ treatment could be predicted. This finding may provide a novel strategy for guiding the development of more personalized medicine for SjD.

## Introduction

Sjögren’s disease (SjD) is a chronic exocrinopathy that leads to salivary and lacrimal gland hypofunction with resultant dry mouth and eye symptoms. SjD is considered to be a systemic autoimmune disease because of the presence of lymphocytic infiltration into affected tissues, serum autoantibodies, and hypergammaglobulinemia [1]. Increased apoptosis of salivary gland epithelial cells as a result of lysosomal dysfunction has been implicated in the development of salivary gland hypofunction and autoimmunity [2–6].

Chloroquine (CQ) and its derivative hydroxychloroquine (HCQ) are lysosomotropic agents, which deactivate lysosomal function by alkalinizing the lysosomal pH. Specifically, they inhibit endolysosomal, autophagic, and endolysosomal toll-like receptor (TLR) pathways [7, 8]. HCQ is frequently prescribed to patients with SjD. Although its efficacy in SjD has been shown in several observational studies in the 1990s [9–12], two more recent randomized controlled trials in SjD patients did not substantiate the expected benefits [13, 14].

Transcriptome analysis of minor salivary glands of SjD patients identified increased expression of *LAMP3* mRNA, encoding lysosome-associated membrane protein 3 (LAMP3), and this expression was associated with some SjD-related clinical findings, such as lymphocytic infiltration, serum autoantibodies and hypergammaglobulinemia [2]. Unlike LAMP1 and LAMP2, which are distributed in many tissues and associated with general lysosomal functions, LAMP3 is expressed in limited several cell types, such as dendritic cells and type II pneumocytes, and involved in their specific functions, including antigen presentation via major histocompatibility complex class II and surfactant synthesis, secretion, and recycling [15]. Confocal immunofluorescent studies on SjD patients’ salivary glands revealed ectopic LAMP3 expression on both lysosomal membrane and plasma membrane of salivary gland epithelial cells as well as in infiltrating immune cells [2, 3]. Interestingly, non-obese diabetic mice, a spontaneous model of SjD, also have increased LAMP3 expression in their salivary glands, and mice in which LAMP3 overexpression was induced in their salivary glands develop an SjD-like phenotype with progressive salivary gland hypofunction, local lymphocytic infiltration, and the presence of serum anti-Ro/SSA and anti-La/SSB antibodies [4]. The underlying mechanism is thought to rely on promotion of apoptosis via induction of lysosomal membrane instability by LAMP3 [2–4].

As patients with SjD present with heterogeneous symptoms, recent studies have focused on stratifying them into homogeneous subsets to develop a personalized treatment strategy [16, 17]. Considering the LAMP3-associated pathophysiology, correction of lysosomal function can be a promising therapeutic approach for SjD. In the current study, we utilized this information to test the hypothesis that targeting patients with elevated salivary gland *LAMP3* expression for HCQ therapy will improve overall clinical outcome of this drug by a retrospective cohort study in combination with *in vitro* and *in vivo* experiments.

## Results

### CQ prevents LAMP3-induced apoptotic cell death via deactivation of cathepsin B

**Fig 1:**
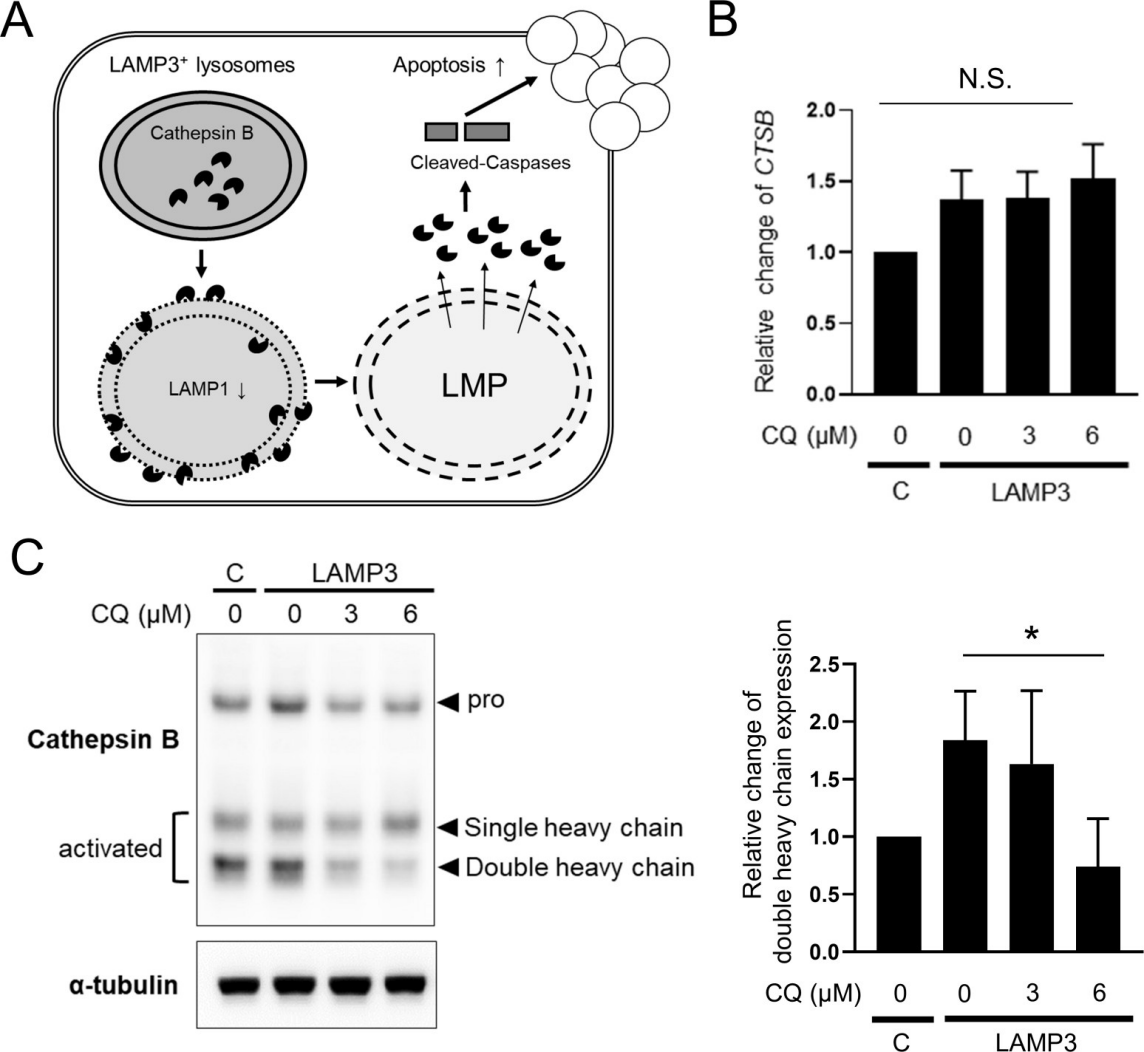
Chloroquine deactivate cathepsin B in LAMP3-overexpressing cells. Stably LAMP3-overexpressing A253 cells were treated with chloroquine (CQ) for 24 hours at increasing concentrations. (**A**) Molecular mechanism about LAMP3-induced apoptotic cell death. (**B**) Relative change in *CTSB* expression (*n* = 4). C: control A253 cells. (**C**) Representative Western blots showing expression of cathepsin B and α-tubulin (control). Bar chart shows relative change of cathepsin B expression normalized by α-tubulin expression compared to control A253 cells (*n* = 3). *P < 0.05, N.S. = not significant (Student’s *t*-tests with Dunnett’s correction).

LAMP3 destabilizes the lysosomal membrane via degradation of LAMP1, which leads to lysosomal membrane permeabilization (LMP), and leakage of activated lysosomal enzymes into the cytoplasm. This in turn activates caspases, resulting in apoptosis. LAMP3-associated LAMP1 degradation and LMP are dependent on cathepsin B activity (**Fig 1A**) [3].

To investigate whether CQ can inhibit LAMP3-induced apoptotic cell death, we first treated stably LAMP3-overexpressing A253 cells with CQ for 24 hours and then analyzed cathepsin B expression, LAMP1 expression, galectin-3 puncta formation (a marker of LMP), and apoptotic cell death. Pro-cathepsin B, which is synthesized on the rough endoplasmic reticulum, matures into single and double heavy chains in the late endosome and lysosome, respectively [18]. Although CQ treatment did not change mRNA expression of *CTSB* (encoding cathepsin B) (**Fig 1B**), it did decrease the amount of the double heavy chain form of cathepsin B (**Fig 1C**), confirming that CQ, an alkalinizing agent, can inhibit the pH-dependent maturation process of cathepsin B in the lysosome [19].

**Fig 2:**
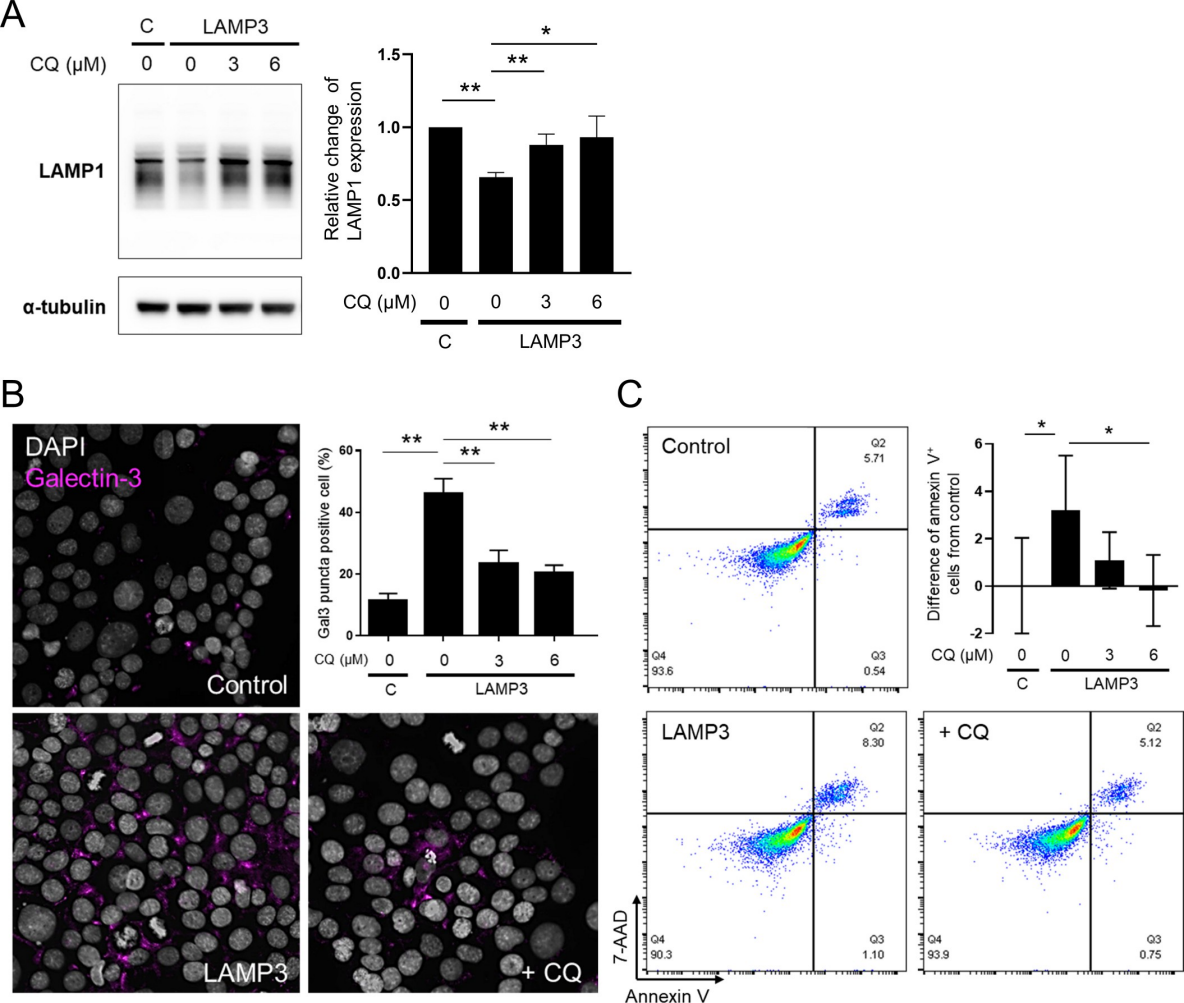
Chloroquine prevents LAMP3-induced apoptotic cell death *in vitro*. Stably LAMP3-overexpressing A253 cells were treated with chloroquine (CQ) for 24 hours at increasing concentrations. (**A**) Representative Western blots showing expression of LAMP1 and α-tubulin (control). Bar chart shows relative change of LAMP1 expression normalized by α-tubulin expression compared to control A253 cells (*n* = 3). C: control A253 cells. (**B**) Representative immunofluorescent images showing staining for galectin-3 (magenta) (40× magnification). Bar chart shows percentage of galectin-3 (Gal3) puncta-positive cells (*n* = 3). (**C**) Number of apoptotic cells was determined by flow cytometry using APC Annexin V/7-AAD. Bar chart shows percentage of difference in Annexin V-positive cells from control (*n* = 5). Values shown are mean ± SD. *P < 0.05, **P < 0.01, N.S. = not significant (Student’s *t*-tests with Dunnett’s correction).

In addition, CQ treatment restored LAMP1 expression (**Fig 2A**), reduced galectin-3 puncta formation (**Fig 2B**), and decreased apoptosis (**Fig 2C**) in LAMP3-overexpressing cells compared to vehicle-treated LAMP3-overexpressing cells. These results demonstrated that CQ decreases LAMP3-induced apoptotic cell death *in vitro* by deactivating cathepsin B.

### HCQ treatment decreases LAMP3-induced apoptotic cell death in mice

**Fig 3:**
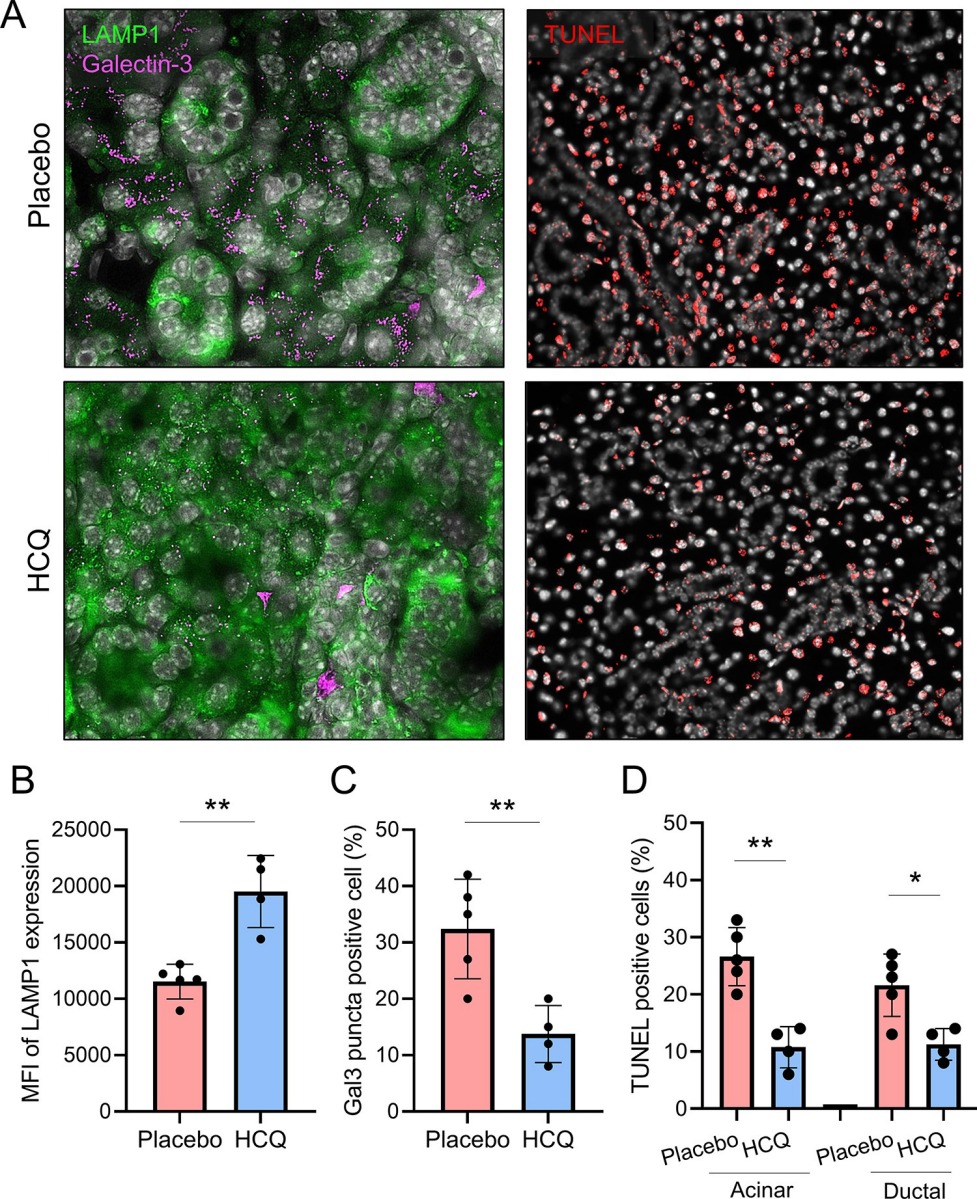
Hydroxychloroquine treatment decreases LAMP3-induced cell death *in vivo*. Submandibular glands of C57BL/6 mice were instilled with AAV2-LAMP3. After 4 months, mice were given weekly intraperitoneal injections of 60 mg/kg HCQ (*n* = 4) or placebo (*n* = 5) for 3 months. (**A**) Representative submandibular gland tissue sections showing staining for LAMP1 (green) and galectin-3 (magenta) (40× magnification) or terminal deoxynucleotidyl transferase dUTP nick-end labeling (TUNEL, red) (20× magnification). (**B**) Bar chart shows mean fluorescence intensity of LAMP1 expression. (**C**) Bar chart shows percentage of galectin-3 (Gal3) puncta-positive cells. (**D**) Bar chart shows percentage of TUNEL-positive epithelial cells. Values shown are mean ± SD. *P < 0.05, **P < 0.01, N.S. = not significant (Student’s *t*-tests).

Next, to examine whether HCQ can decrease LAMP3-induced apoptotic cell death *in vivo*, we induced epithelial LAMP3 overexpression in the submandibular glands of female, 2-month-old C57BL/6 mice by retroductal cannulation with adeno-associated virus serotype 2 vectors encoding the gene for LAMP3 (AAV2-LAMP3), as previously reported [4]. After 4 months—when all mice have established SjD-like disease—mice were treated with weekly intraperitoneal injections of HCQ or placebo for 3 months. At the end of the study, the submandibular glands were collected and analyzed for changes in apoptosis and histology. Consistent with the above *in vitro* results, HCQ treatment restored LAMP1 expression (**Fig 3A** and **3B**) and reduced galectin-3 puncta formation (**Fig 3A** and **3C**) in salivary glands of LAMP3-overexpressing mice. Terminal deoxynucleotidyl transferase dUTP nick-end labeling (TUNEL) assay for staining cells with damaged DNA and undergoing apoptosis showed that the number of apoptotic epithelial cells was significantly decreased in the HCQ-treated mice compared to placebo-treated mice (**Fig 3A** and **3D**).

**Fig 4:**
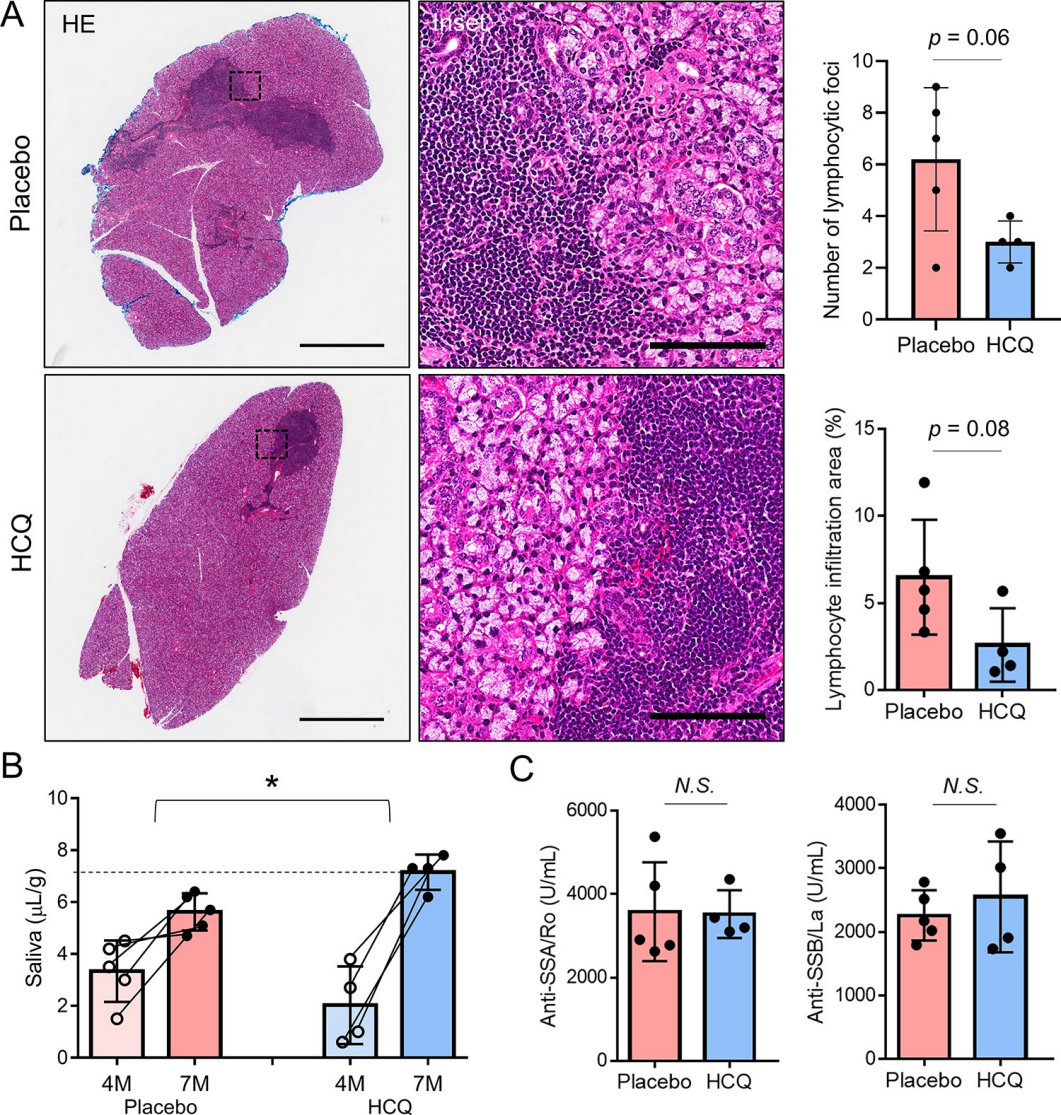
Hydroxychloroquine treatment ameliorates LAMP3-associated sialadenitis *in vivo*. Submandibular glands of C57BL/6 mice were instilled with AAV2-LAMP3. After 4 months, mice were given weekly intraperitoneal injections of 60 mg/kg HCQ (*n* = 4) or placebo (*n* = 5) for 3 months. (**A**) Representative submandibular gland tissue sections showing hematoxylin & eosin (HE) staining. Scale bars = 1 mm (inset: 100 μm). Bar charts shows the number of lymphocytic foci and the average size of lymphocytic infiltration areas in both the submandibular glands of each mouse. (**B**) Graph showing pilocarpine-stimulated salivary flow rate per body weight in 20 minutes. The dashed line indicates a reference level of salivary flow rate derived from control mice (instilled with AAV2-GFP). (**C**) Bar charts showing serum anti-Ro/SSA or anti-La/SSB antibody levels. Values shown are mean ± SD. *P < 0.05, N.S. = not significant (Student’s *t*-tests).

Histological analysis of the glands suggested a trend towards decreased lymphocytic infiltration in the HCQ-treated mice (**Fig 4A**). Treatment with HCQ resulted in a significant increase in salivary flow rate compared to placebo (**Fig 4B**), while serum anti-Ro/SSA and anti-La/SSB antibody levels were not affected (**Fig 4C**). In short, these results suggested that HCQ treatment decreased LAMP3-induced apoptotic cell death and restores salivary gland function in a mouse model of SjD.

### Clinical response to HCQ in SjD patients is associated with salivary gland LAMP3 mRNA expression level

**Fig 5:**
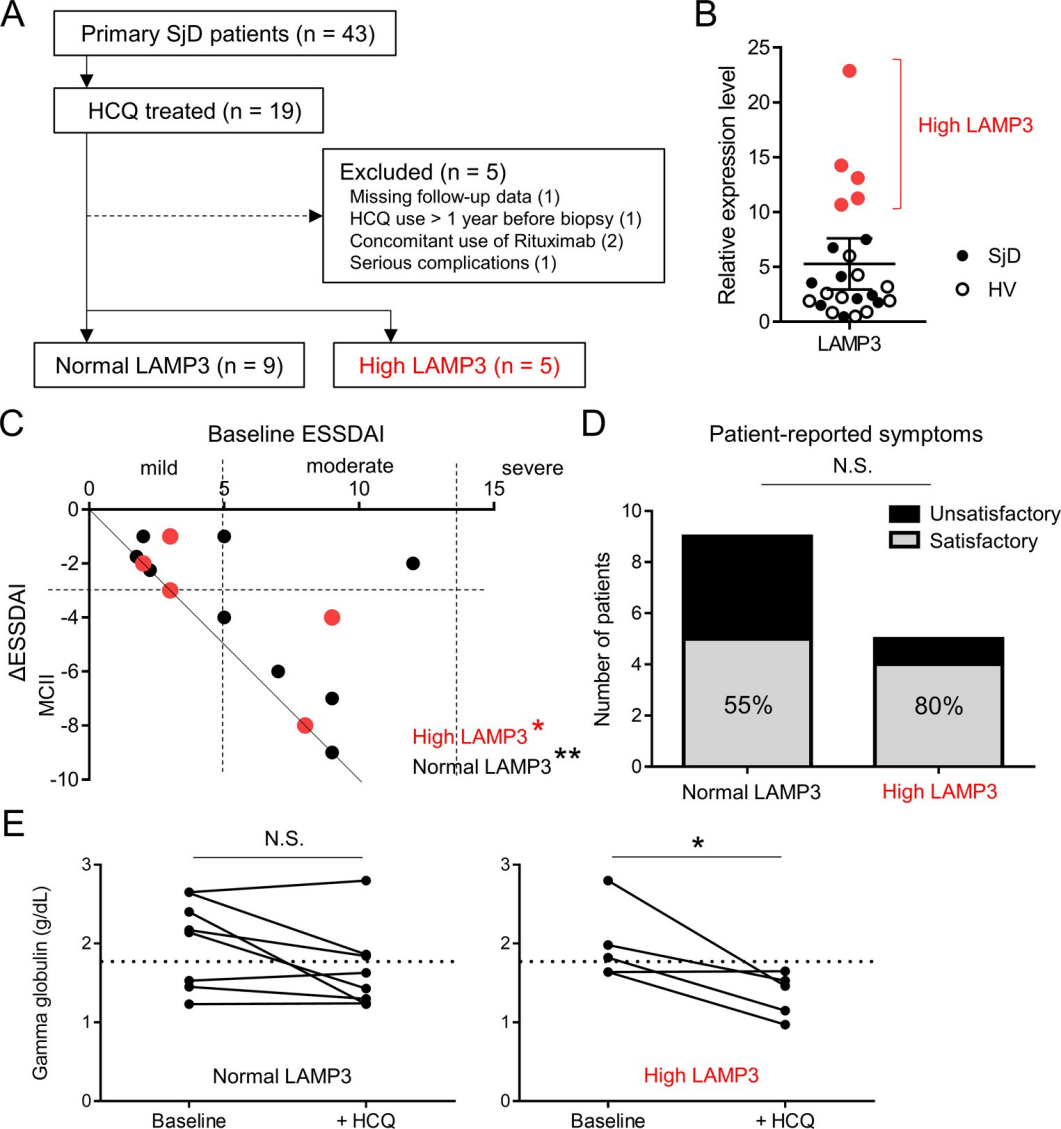
Clinical response to hydroxychloroquine in SjD patients is associated with salivary gland *LAMP3* mRNA expression level. (**A**) Flow diagram showing SjD patient selection. (**B**) Dot plot showing relative expression of *LAMP3* with mean and 95% confidence interval in labial minor salivary glands from SjD patients (*n* = 14) and healthy volunteers (HV, *n* = 10). (**C**) Dot plot showing baseline ESSDAI and change in ESSDAI after HCQ treatment. ΔESSDAI ≥ 3 is considered as minimal clinically important improvement (MCII). Continuous line represents complete response (ESSDAI = 0) to HCQ treatment. (**D**) Bar chart showing patient-reported symptoms after HCQ treatment. ESSPRI < 5 is considered as satisfactory control of SjD symptoms. (**E**) Graphs showing serum gamma globulin levels before and after HCQ treatment. Dotted lines show upper limit of normal range. *P < 0.05, **P < 0.01, N.S. = not significant (paired Student’s t-test for quantitative variables or Chi-squared test for categorical variables).

Our *in vitro* and *in vivo* experiments showed that CQ/HCQ can reduce LAMP3-induced apoptotic cell death. Finally, to confirm HCQ’s effect on LAMP3-induced changes in SjD patients, we retrospectively analyzed the efficacy of HCQ treatment in a cohort of SjD patients seen at the Clinical Hospital of the Medical School of Ribeirão Preto in São Paulo, Brazil. For this analysis, a cohort of 43 SjD patients were selected. Of the 43 patients, 19 had been treated with HCQ. We excluded 5 patients because of missing follow-up data, HCQ use more than 1 year before labial minor salivary gland biopsy, concomitant use of rituximab, or serious complications, such as new diagnosis of cancer (**Fig 5A**). For comparison, a group of 10 age-matched healthy volunteers were recruited from the same hospital.

RNA-sequencing data showed that 5 SjD patients had a high *LAMP3* level (above mean and 95% confidence interval) and 9 showed a normal *LAMP3* level, comparable with that of the healthy volunteers (**Fig 5B**). At baseline (before HCQ treatment), there were no significant differences in patient characteristics between the high-*LAMP3* and normal-*LAMP3* groups. But, the proportion of patients with a high lymphocytic focus score (≥ 3) was higher in the high-*LAMP3* group (80% vs. 44%, *P* = 0.20), while the proportion of patients with a decreased salivary flow rate (≤ 0.1 mL/min) was higher in the normal-*LAMP3* group (20% vs. 56%, *P* = 0.20). Most of the patients were concomitantly treated with low-dose corticosteroids (prednisolone-equivalent: < 10 mg/day) and/or methotrexate (**Table 1**).

**Table 1:** Baseline profile of SjD patients treated with hydroxychloroquine, stratified by minor salivary gland *LAMP3* mRNA expression.

*LAMP3* mRNA expression	High (*n* = 5)	Normal (*n* = 9)	*P* value
Age, years	47 ± 13	49 ± 12	0.81
Female	5 (100%)	9 (100%)	-
Anti-SSA/SSB antibody positivity	5 (100%)	8 (89%)	0.44
Focus score ≥ 3	4 (80%)	4 (44%)	0.20
Salivary flow rate ≤ 0.1 mL/min	1 (20%)	5 (56%)	0.20
Schirmer test ≤ 5mm/5min	3 (60%)	6 (67%)	0.80
Extraglandular manifestations	4 (80%)	6 (67%)	0.60
Fever/weight loss	0	1	
Arthritis/arthralgia	3	4	
Lymphadenopathy	2	0	
Purpura	0	2	
nterstitial lung disease	0	1	
ESSDAI	5.0 ± 2.9	5.9 ± 3.4	0.66
Gamma globulin, g/dL	1.98 ± 0.48	1.99 ± 0.50	0.65
Concomitant treatment	4 (80%)	7 (78%)	0.92
Corticosteroid	2	6	
Methotrexate	3	4	

The median duration of HCQ treatment was 2 years in the high-*LAMP3* group and 4 years in the normal-*LAMP3* group. The European League against Rheumatism Sjögren’s syndrome disease activity index (ESSDAI) was significantly decreased after HCQ treatment in both the high-*LAMP3* group (5.0 ± 2.9 to 1.4 ± 2.2, *P* < 0.05) and normal-*LAMP3* group (5.9 ± 3.4 to 2.2 ± 3.2, *P* < 0.01). However, of the 8 patients with moderate disease activity (ESSDAI: 5–13 [20]) at baseline, 2/2 patients (100%) in the high-*LAMP3* group and 4/6 patients (67%) in the normal-*LAMP3* group showed minimal clinically important improvement (i.e., change in ΔESSDAI ≥ 3 [21]). Furthermore, 3/5 patients (60%) patients in the high-*LAMP3* group achieved an ESSDAI of 0 after HCQ treatment, while the same was true for 3/9 patients (33%) in the normal-*LAMP3* group (**Fig 5C**). After HCQ treatment, 4/5 patients (80%) in the high-*LAMP3* group and 5/9 patients (55%) in the normal-*LAMP3* group reported satisfactory control of SjD symptoms such as dryness, pain, and fatigue (i.e., the European League against Rheumatism Sjögren’s syndrome patient-reported index (ESSPRI) < 5 [21]) (**Fig 5D**). Analysis of clinical laboratory data showed that HCQ treatment significantly decreased serum gamma globulin levels in the high-*LAMP3* group (1.98 ± 0.48 to 1.35 ± 0.28, *P* < 0.05) but not in the normal-*LAMP3* group (1.99 ± 0.50 to 1.67 ± 0.52, *P* = 0.07). Hypergammaglobulinemia (i.e., serum gamma globulin level > 1.7 g/dL) was improved in 3/3 patients (100%) in the high-*LAMP3* group and 2/5 (40%) in the normal-*LAMP3* group after HCQ treatment (**Fig 5E**). Severe adverse events related to HCQ use were not reported during the observation period.

Taken together, these results suggested that HCQ treatment can decrease the severity of SjD symptoms associated with high salivary gland *LAMP*3 expression in agreement with those of our *in vitro* and *in vivo* experiments.

## Discussion

HCQ has been used for many years to treat various autoimmune diseases, especially systemic lupus erythematosus and rheumatoid arthritis [22]. In this context, HCQ has been considered as an immunomodulator that regulates immune cells by impairing endolysosomal TLR pathways [23, 24]. Ours is the first study that focused on the effect of HCQ on salivary gland epithelial cells specifically in autoimmune sialadenitis. In this study, we demonstrated that HCQ decreased apoptotic cell death induced by LAMP3 overexpression in salivary gland epithelial cells. In our mouse experiment, administration of HCQ ameliorated the SjD-like phenotype induced by LAMP3 overexpression. Furthermore, in our cohort of SjD patients, HCQ treatment showed a larger decrease in the ESSDAI, ESSPRI, and serum gamma globulin levels in the patients with high *LAMP3* expression compared to those with normal *LAMP3* expression.

Our results support the need for better molecular characterization of SjD to understand the etiology of the disease and to better personalize treatment options for patients. Recently, various methods for clustering or subgrouping SjD patients have been reported [16, 17]; however, it is still unknown which method is appropriate and meaningful in real-world clinical practice. Compared to existing methods, our subgrouping based on salivary gland *LAMP3* mRNA expression is practical because this level can be easily evaluated by real-time reverse transcription PCR analysis of labial minor salivary gland biopsies, which are commonly performed during diagnostic evaluation for SjD. The determination of LAMP3 protein expression by immunostaining of salivary gland biopsies may provide more direct evidence, although the staining and quantification methods need to be standardized.

HCQ is known as a toxic agent that inhibits the physiological lysosomal trafficking and degradation system and induces apoptotic cell death in a dose-dependent manner [25, 26]. Although the safe dosage and administration of this old drug have been established in a long clinical history, rare but serious risks have been associated with HCQ use, including cardiac and retinal toxicity [27]. Interestingly, our results indicated that HCQ paradoxically prevented apoptosis in LAMP3-overexpressing epithelial cells. Presumably, HCQ balances the lysosomal dysfunction in LAMP3-overexpressing cells, while impairing the lysosomal system in normal cells, making it a good candidate for a personalized strategy in SjD patients with high *LAMP3* expression.

Although both the lacrimal and salivary glands are targeted in SjD, the effect of HCQ on lacrimal glands was not investigated in this study. A recent study showed that *LAMP3* expression was upregulated in conjunctiva of SjD patients, and the expression levels were significantly correlated with the severity of dry eye symptoms [28], suggesting that LAMP3 overexpression is associated with the development of dacryoadenitis as well as sialadenitis. Future studies need to clarify if HCQ can ameliorate LAMP3-associated dacryoadenitis.

Limitations of this study are limited statistical analysis due to the small sample size of our patient cohort and the potential for bias in selection and reporting because of the retrospective design. We acknowledge the absence of statistically significant differences in ESSDAI and ESSPRI changes by HCQ treatment between the high-*LAMP3* and normal-*LAMP3* group. The study also lacked the evaluation of salivary flow rate in patients to validate the finding that HCQ treatment restored the salivary flow rate in mice. In addition, we still have no direct evidence on association between LAMP3 expression and salivary gland hypofunction in SjD patients beyond the mouse data. Previous studies suggest SjD patient’s salivary flow rate is a response to various conditions, including lymphocytic infiltration, atrophy and fibrosis of the glands [29, 30]. Further research in a larger set of SjD patients is required to statistically verify our findings with corrections for confounding factors, such as baseline disease activity and concomitant medications.

In conclusion, we have shown that HCQ can ameliorate LAMP3-associated salivary gland hypofunction and our pilot analysis on the patients’ data suggested that high *LAMP3* expression could be used as a biomarker to stratify SjD patients who respond adequately to HCQ treatment. Determining the salivary gland *LAMP3* level prior to initiating HCQ treatment may therefore provide a novel strategy for guiding personalized medicine for SjD.

## Materials and methods

### Human subjects

We included the consecutive patients who visited the Clinical Hospital of the Medical School of Ribeirão Preto of the University of São Paulo, underwent labial minor salivary gland biopsies between 2013 and 2016, and met the 2002 American-European Consensus Group criteria [31] and age-matched healthy volunteers. Labial minor salivary gland biopsies were surgically excised from all subjects for bulk RNA sequencing and histological evaluation to determine lymphocytic focus score. RNA sequencing libraries were prepared as described previously [4], and the raw data files were deposited in the National Center of Biotechnology Information Gene Expression Omnibus database (GSE154926). Clinical symptom data were collected using standardized questionnaires and physical examinations at each visit. Data regarding laboratory measures, such as serum anti-Ro/SSA antibody, anti-La/SSB antibody and gamma globulin levels, were obtained from clinically ordered tests.

All clinical investigations were conducted in accordance with the Declaration of Helsinki principles and approved by the Brazilian Committee of Ethics in Research (37688914.2.0000.5440). All subjects provided written informed consent prior to the initiation of any study procedure.

### Animals

LAMP3-overexpressing mice were established as described previously [4]. Briefly, AAV2-LAMP3 were delivered to both submandibular glands (10^11^ particles/mouse in 100 μL) of female, 2-month-old C57BL/6 mice (Charles River Laboratories, USA) by retrograde ductal instillation through a thin cannula. After 4 months, mice were randomly assigned to weekly intraperitoneal HCQ injections (Plaquenil® tablet (Sanofi, USA) in a vehicle of phosphate buffered saline (PBS) at 60 mg/kg or placebo (PBS only) for 3 months, based on a previous report [32]. Pilocarpine-stimulated salivary flow rate in 20 minutes was determined immediately prior to initiation of HCQ treatment and after 3 months.

At the end of the study, mice were sacrificed and blood and whole submandibular glands were collected. Serum was separated by centrifugation and then stored at -80°C. Presence of serum anti-Ro/SSA and anti-La/SSB antibodies were tested using the Mouse Anti-SSA/Ro60 Ig’s (total) ELISA Kit (#5710) and Mouse Anti-SSB Ig’s (total (A+G+M) ELISA Kit (#5810), respectively, according to the manufacturer’s instructions (both purchased from Alpha Diagnostic International, USA). Lymphocytic infiltration in both the submandibular glands were investigated using hematoxylin & eosin staining. The outcomes were evaluated by unblinded researchers.

All procedures involving live animals were approved based on institutional guidelines and standard operating procedures following the NIH Guide for the Care and Use of Laboratory Animals (approval number: 18–863).

### Cells

A253 cells (ATCC, USA)—a human salivary gland cell line—were cultured in McCoy’s 5A Medium (Thermo Fisher Scientific, USA) supplemented with 10% fetal bovine serum. Stably LAMP3-overexpressing and control A253 cells were established as described previously [3]. All cells were incubated at 37°C with humidity and 5% CO_2_.

### Quantitative real-time reverse transcription PCR

Total RNA was extracted from A253 cells using the RNeasy Mini Kit (QIAGEN, USA), treated with RNase-Free DNase Set (QIAGEN), and reverse transcribed into cDNA using the SuperScript VILO cDNA Synthesis Kit (Thermo Fisher Scientific). To calculate transcript expression levels, TaqMan gene expression assays for *CTSB* (Hs00947439_m1) and *ACTB* (Hs01060665_g1) were used (Thermo Fisher Scientific). Gene expression relative to *ACTB* was calculated using the *ΔΔ*Ct method.

PCR cycles were performed using the Quantstudio3 Real-Time PCR System (Life Technologies, USA) with the following conditions: 2 minutes at 50°C, 10 minutes at 95°C, 50 cycles of 15 seconds at 95°C, and 1 minute at 60°C.

### Western blotting

A253 cells were lysed in RIPA Lysis and Extraction Buffer with protease and phosphatase inhibitors (all from Thermo Fisher Scientific) and cleared by centrifugation at 17,000 g at 4°C for 25 minutes. Supernatants were heated at 97°C in NuPAGE LDS Sample Buffer for 10 minutes, resolved by SDS-PAGE, and electrophoretically transferred to polyvinylidene difluoride membranes (all from Thermo Fisher Scientific). Membranes were blocked with 2% non-fat dried milk at 25°C for 1 hour, and then incubated at 4°C overnight with one of the following primary antibodies: anti-cathepsin B (#AF953, R&D system, USA), anti-LAMP1 (#21997-1-AP, Proteintech, USA) or anti-α-tubulin (#T6199, Sigma-Aldrich, USA). After washing three times, membranes were incubated with rabbit or mouse IgG horseradish peroxidase-linked whole antibody (Sigma-Aldrich) at 25°C for 1 hour. Signals were visualized using Super Signal West Pico Chemiluminescent Substrate or Super Signal West Pico PLUS Chemiluminescent Substrate (both from Thermo Fisher Scientific).

### Galectin-3 puncta assay

A253 cells were fixed with 4% paraformaldehyde for 15 minutes, permeabilized using 0.1% Triton-X-100 (Sigma-Aldrich) for 10 minutes and blocked with 2% bovine serum albumin (BSA) for 30 minutes, all at room temperature. Then, cells were incubated with a mixture of 10 μg/mL mouse anti-galectin-3 antibody (#ab2785, Abcam, USA) in 2% BSA at 4°C overnight. The next day, cells were incubated with a mixture of 10 μg/mL Alexa Fluor-647 anti-mouse IgG (Jackson ImmunoResearch Laboratories, Inc., USA) in 2% BSA at room temperature for 1 hour.

Formalin-fixed paraffin embedded sections of murine submandibular glands were deparaffinized, rehydrated and subjected to citric acid microwave antigen retrieval. Slides were blocked with 2% BSA (Sigma-Aldrich) and permeabilized by 0.1% Triton-X-100 (Sigma-Aldrich) for 30 minutes at 25°C. Slides were incubated with mouse anti-galectin-3 (#ab2785, Abcam) and rabbit anti-LAMP1 (#21997-1-AP, Proteintech) antibodies at 4°C overnight, followed by incubation with Alexa Fluor-647 anti-mouse IgG and Alexa Fluor-488 anti-rabbit IgG (Jackson ImmunoResearch Laboratories, Inc.) at room temperature for 1 hour.

Sections were subsequently counterstained with DAPI (#ab104139, Abcam). Images were acquired using a fluorescent microscope (Nikon, Japan) and analyzed using ImageJ software (public domain, source: National Institutes of Health, USA).

### Apoptosis assay

To detect apoptosis *in vitro*, A253 cells were stained using the APC Annexin V Apoptosis Detection Kit with 7-AAD (#640930, BioLegend, USA), and apoptotic cells were detected using the BD Accuri Flow Cytometer (BD Biosciences, USA).

Apoptosis *in vivo* was visualized using a TUNEL assay (#ab66110, Abcam) on formalin-fixed, paraffin-embedded murine submandibular gland sections, according to the manufacturer’s instructions. After counterstaining with DAPI mounting medium (Abcam), images were acquired with a fluorescent microscope (Nikon) and analyzed using ImageJ software.

### Statistical analysis

Data are presented as mean ± standard deviation (SD). Quantitative variables were compared using a two-tailed paired or unpaired Student’s *t*-test. When appropriate, Dunnett’s correction was used to correct for multiple hypothesis testing. Categorical variables were compared using the Chi-squared test. *P* values < 0.05 were considered statistically significant. All analyses were performed using GraphPad Prism 8.0 software.

## Supporting information

S1 File (PDF)

## References

[1] Odani T, Chiorini JA. Targeting primary Sjögren’s syndrome. Mod Rheumatol 2019;29:70–86. 30424703 10.1080/14397595.2018.1546268

[2] Tanaka T, Warner BM, Odani T, Ji Y, Mo YQ, Nakamura H, et al. LAMP3 induces apoptosis and autoantigen release in Sjögren’s syndrome patients. Sci Rep 2020;10:15169. 32939030 10.1038/s41598-020-71669-5 PMC7494869

[3] Tanaka T, Warner BM, Michael DG, Nakamura H, Odani T, Yin H, et al. LAMP3 inhibits autophagy and contributes to cell death by lysosomal membrane permeabilization. Autophagy 2022;18:1629–1647. doi: 10.1080/15548627.2021.1995150 34802379 PMC9298453

[4] Nakamura H, Tanaka T, Pranzatelli T, Ji Y, Yin H, Perez P, et al. Lysosome-associated membrane protein 3 misexpression in salivary glands induces a Sjögren’s syndrome-like phenotype in mice. Ann Rheum Dis 2021;80:1031–1039. 33658234 10.1136/annrheumdis-2020-219649 PMC8292598

[5] Mo YQ, Nakamura H, Tanaka T, Odani T, Perez P, Ji Y, et al. Lysosomal exocytosis of HSP70 stimulates monocytic BMP6 expression in Sjögren’s syndrome. J Clin Invest 2022;132:e152780. 35113815 10.1172/JCI152780 PMC8920330

[6] Manganelli P, Fietta P. Apoptosis and Sjögren syndrome. Semin Arthritis Rheum 2003;33:49–65. 12920696 10.1053/sarh.2003.50019

[7] Chandler LC, Yusuf IH. Immunomodulatory Effects of Hydroxychloroquine and Chloroquine in Viral Infections and Their Potential Application in Retinal Gene Therapy. Int J Mol Sci 2020;21:4972. doi: 10.3390/ijms21144972 32674481 PMC7404262

[8] Mauthe M, Orhon I, Rocchi C, Zhou X, Luhr M, Hijlkema KJ, et al. Chloroquine inhibits autophagic flux by decreasing autophagosome-lysosome fusion. Autophagy 2018;14:1435–55. doi: 10.1080/15548627.2018.1474314 29940786 PMC6103682

[9] Fox RI, Chan E, Benton L, Fong S, Friedlaender M, Howell FV. Treatment of primary Sjögren’s syndrome with hydroxychloroquine. Am J Med 1988;85:62–7. 3177432 10.1016/0002-9343(88)90365-8

[10] Kruize AA, Hené RJ, Kallenberg CG, van Bijsterveld OP, van der Heide A, Kater L, et al. Hydroxychloroquine treatment for primary Sjögren’s syndrome: a two year double blind crossover trial. Ann Rheum Dis 1993;52:360–4. 8323383 10.1136/ard.52.5.360 PMC1005050

[11] Fox RI, Dixon R, Guarrasi V, Krubel S. Treatment of primary Sjögren’s syndrome with hydroxychloroquine: a retrospective, open-label study. Lupus 1996;5 Suppl 1:S31–6. 8803908

[12] Tishler M, Yaron I, Shirazi I, Yaron M. Hydroxychloroquine treatment for primary Sjögren’s syndrome: its effect on salivary and serum inflammatory markers. Ann Rheum Dis 1999;58:253–6. 10364906 10.1136/ard.58.4.253 PMC1752865

[13] Gottenberg JE, Ravaud P, Puéchal X, Le Guern V, Sibilia J, Goeb V, et al. Effects of hydroxychloroquine on symptomatic improvement in primary Sjögren syndrome: the JOQUER randomized clinical trial. Jama 2014;312:249–58. 25027140 10.1001/jama.2014.7682

[14] Yoon CH, Lee HJ. Effect of Hydroxychloroquine Treatment on Dry Eyes in Subjects with Primary Sjögren’s Syndrome: a Double-Blind Randomized Control Study. J Korean Med Sci 2016;31:1127–35. 27366013 10.3346/jkms.2016.31.7.1127 PMC4901007

[15] Alessandrini F, Pezzè L, Ciribilli Y. LAMPs: Shedding light on cancer biology. Semin Oncol 2017;44:239–53. doi: 10.1053/j.seminoncol.2017.10.013 29526252

[16] Soret P, Le Dantec C, Desvaux E, Foulquier N, Chassagnol B. A new molecular classification to drive precision treatment strategies in primary Sjögren’s syndrome. Nat Commun 2021;12:3523. 34112769 10.1038/s41467-021-23472-7 PMC8192578

[17] Collins A, Lendrem D. Revisiting the JOQUER trial: stratification of primary Sjögren’s syndrome and the clinical and interferon response to hydroxychloroquine. Rheumatol Int 2021;41:1593–600. 34165604 10.1007/s00296-021-04927-y PMC8316226

[18] Cavallo-Medved D, Sloane BF, Moin K. Cathepsin B. In: Choi S, editor. Encyclopedia of Signaling Molecules. New York, NY: Springer New York; 2017. p. 1–17.

[19] Mijanović O, Branković A, Panin AN, Savchuk S, Timashev P, Ulasov I, et al. Cathepsin B: A sellsword of cancer progression. Cancer Lett 2019;449:207–14. doi: 10.1016/j.canlet.2019.02.035 30796968 PMC6488514

[20] Seror R, Bowman SJ, Brito-Zeron P, Theander E, Bootsma H, Tzioufas A, et al. EULAR Sjögren’s syndrome disease activity index (ESSDAI): a user guide. RMD Open 2015;1:e000022. 26509054 10.1136/rmdopen-2014-000022 PMC4613159

[21] Seror R, Bootsma H, Saraux A, Bowman SJ, Theander E, Brun JG. Defining disease activity states and clinically meaningful improvement in primary Sjögren’s syndrome with EULAR primary Sjögren’s syndrome disease activity (ESSDAI) and patient-reported indexes (ESSPRI). Ann Rheum Dis 2016;75:382–9. 25480887 10.1136/annrheumdis-2014-206008

[22] Dima A, Jurcut C, Arnaud L. Hydroxychloroquine in systemic and autoimmune diseases: Where are we now? Joint Bone Spine 2021;88:105143. doi: 10.1016/j.jbspin.2021.105143 33515791

[23] Sacre K, Criswell LA, McCune JM. Hydroxychloroquine is associated with impaired interferon-alpha and tumor necrosis factor-alpha production by plasmacytoid dendritic cells in systemic lupus erythematosus. Arthritis Res Ther 2012;14:R155. doi: 10.1186/ar3895 22734582 PMC3446541

[24] Gardet A, Pellerin A, McCarl CA, Diwanji R, Wang W, Donaldson D, et al. Effect of in vivo Hydroxychloroquine and ex vivo Anti-BDCA2 mAb Treatment on pDC IFNα Production From Patients Affected With Cutaneous Lupus Erythematosus. Front Immunol 2019;10:275. 30846987 10.3389/fimmu.2019.00275 PMC6394354

[25] Chen J, Pan Q, Bai Y, Chen X, Zhou Y. Hydroxychloroquine Induces Apoptosis in Cholangiocarcinoma via Reactive Oxygen Species Accumulation Induced by Autophagy Inhibition. Front Mol Biosci 2021;8:720370. doi: 10.3389/fmolb.2021.720370 34568426 PMC8462510

[26] Kim WU, Yoo SA, Min SY, Park SH, Koh HS, Song SW, et al. Hydroxychloroquine potentiates Fas-mediated apoptosis of rheumatoid synoviocytes. Clin Exp Immunol 2006;144:503–11. doi: 10.1111/j.1365-2249.2006.03070.x 16734620 PMC1941983

[27] Desmarais J, Rosenbaum JT. American College of Rheumatology White Paper on Antimalarial Cardiac Toxicity. Arthritis Rheumatol 2021;73:2151–60. doi: 10.1002/art.41934 34697918

[28] de Paiva CS, Trujillo-Vargas CM, Schaefer L, Yu Z, Britton RA, Pflugfelder SC. Differentially Expressed Gene Pathways in the Conjunctiva of Sjögren Syndrome Keratoconjunctivitis Sicca. Front Immunol 2021;12:702755. 34349764 10.3389/fimmu.2021.702755 PMC8326832

[29] Bookman AA, Shen H, Cook RJ, Bailey D, McComb RJ, Rutka JA, et al. Whole stimulated salivary flow: correlation with the pathology of inflammation and damage in minor salivary gland biopsy specimens from patients with primary Sjögren’s syndrome but not patients with sicca. Arthritis Rheum 2011;63:2014–20. 21337320 10.1002/art.30295

[30] Yin H, Pranzatelli TJF, French BN, Zhang N, Warner BM, Chiorini JA. Sclerosing Sialadenitis Is Associated With Salivary Gland Hypofunction and a Unique Gene Expression Profile in Sjögren’s Syndrome. Front Immunol 2021;12:699722. 34400910 10.3389/fimmu.2021.699722 PMC8363566

[31] Vitali C, Bombardieri S, Jonsson R, Moutsopoulos HM, Alexander EL, Carsons SE, et al. Classification criteria for Sjögren’s syndrome: a revised version of the European criteria proposed by the American-European Consensus Group. Ann Rheum Dis 2002;61:554–8. 12006334 10.1136/ard.61.6.554 PMC1754137

[32] Ruiz A, Rockfield S, Taran N, Haller E, Engelman RW, Flores I, et al. Effect of hydroxychloroquine and characterization of autophagy in a mouse model of endometriosis. Cell Death Dis 2016;7:e2059. doi: 10.1038/cddis.2015.361 26775710 PMC4816166

