# Supplementary material for: Salivary gland LAMP3 mRNA expression is a possible predictive marker in the response to hydroxychloroquine in Sjögren’s disease
Source: PLoS One. 2023 Feb 23;18(2):e0282227. doi: 10.1371/journal.pone.0282227 (PMC9949663; doi:10.1371/journal.pone.0282227)

Original uncropped and unadjusted images for Figure 1C

Cathepsin B

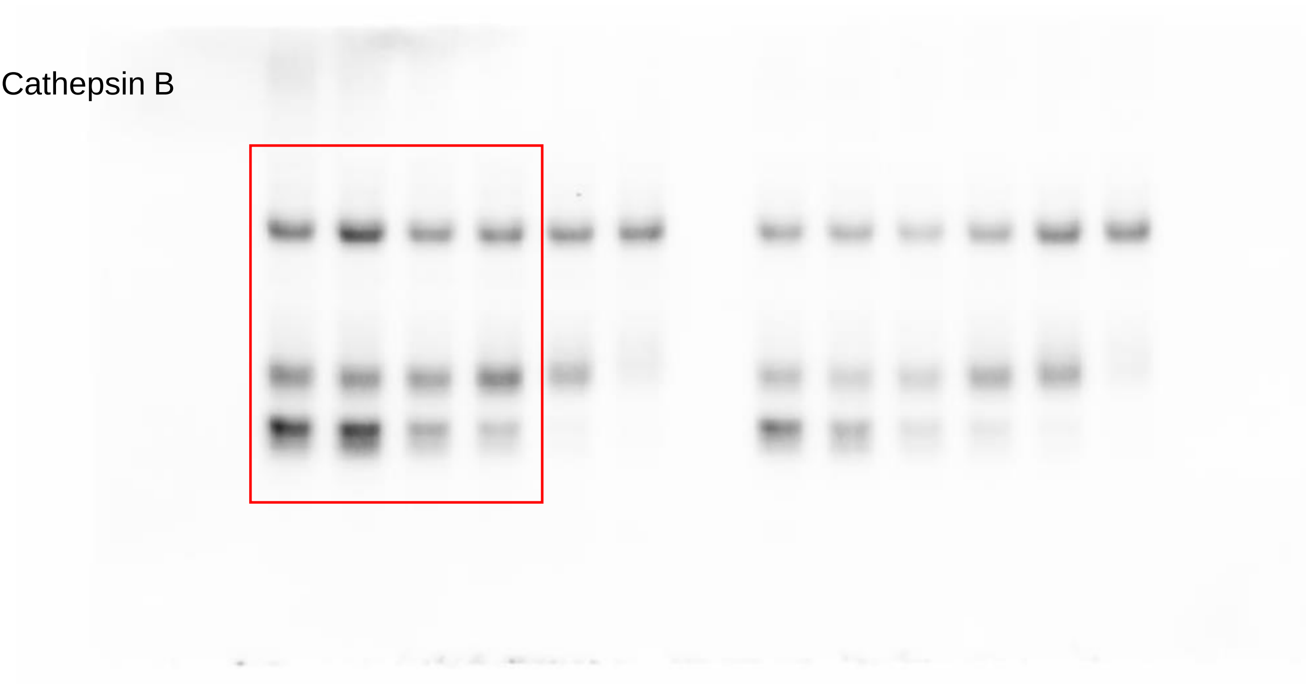

α-tubulin

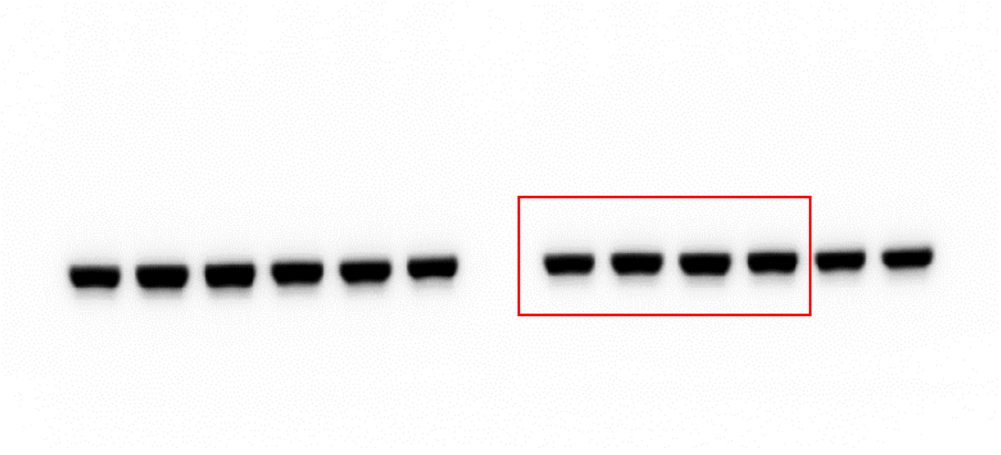

Original uncropped and unadjusted images for Figure 2A

LAMP1

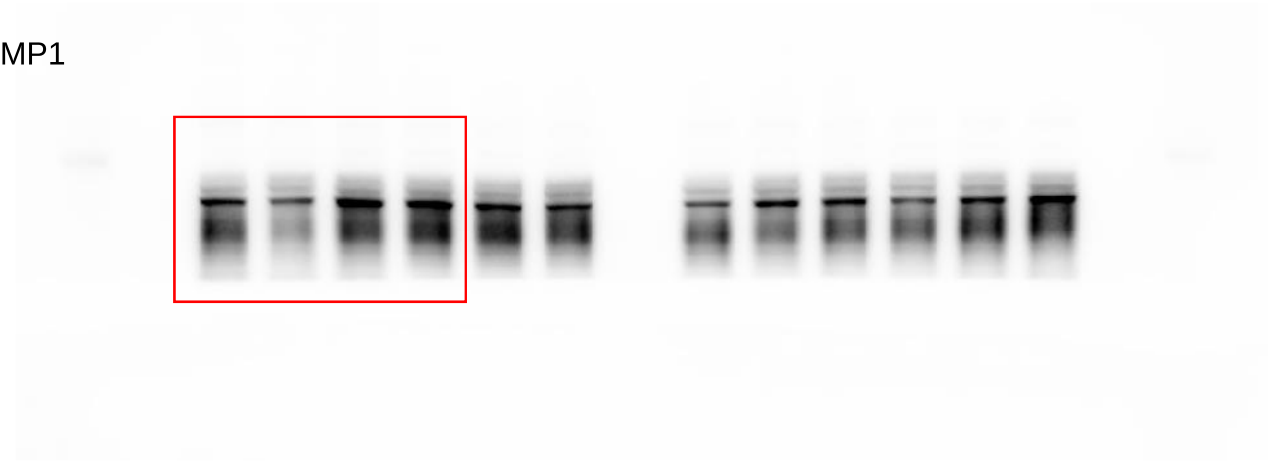

α-tubulin

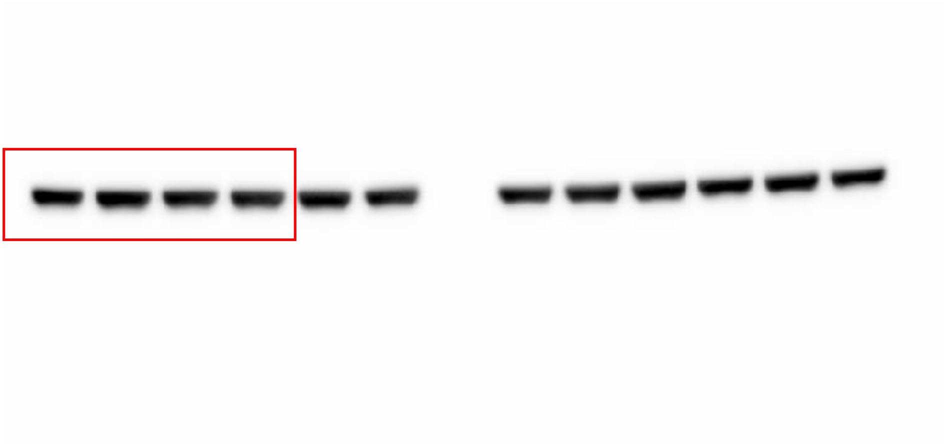

Supplement: S1 File — (PDF) [file pone.0282227.s001.pdf]
